# Regulation of Rac1 Activation in Choroidal Endothelial Cells: Insights into Mechanisms in Age-Related Macular Degeneration

**DOI:** 10.3390/cells10092414

**Published:** 2021-09-14

**Authors:** Aniket Ramshekar, Haibo Wang, M. Elizabeth Hartnett

**Affiliations:** Department of Ophthalmology, John A. Moran Eye Center, University of Utah, 65 Mario Capecchi Dr, Salt Lake City, UT 84132, USA; u1088294@utah.edu (A.R.); haibo.wang@hsc.utah.edu (H.W.)

**Keywords:** age-related macular degeneration, macular neovascularization, choroidal endothelial cells, rho gtpases

## Abstract

Age-related macular degeneration (AMD) is one of the leading causes of blindness worldwide. Vision loss from the neovascular form is associated with the invasion of choroidal endothelial cells into the neural retina to form vision-threatening macular neovascularization (MNV). Anti-angiogenic agents are the current standard of care but are effective in only ~50% of AMD cases. The molecular mechanisms involved in invasive MNV point to the importance of regulating signaling pathways that lead to pathologic biologic outcomes. In studies testing the effects of AMD-related stresses, activation of the Rho GTPase, Rac1, was found to be important for the choroidal endothelial cell invasion into the neural retina. However, current approaches to prevent Rac1 activation are inefficient and less effective. We summarize active Rac1-mediated mechanisms that regulate choroidal endothelial cell migration. Specifically, we discuss our work regarding the role of a multidomain protein, IQ motif containing GTPase activating protein 1 (IQGAP1), in sustaining pathologic Rac1 activation and a mechanism by which active Rap1, a Ras-like GTPase, may prevent active Rac1-mediated choroidal endothelial cell migration.

## 1. Introduction

Age-related macular degeneration (AMD) is one of the leading causes of blindness worldwide [1]. Vision loss occurs in the advanced forms, known as atrophic or neovascular AMD. However, early and intermediate AMD often manifest before symptoms are noted and progress to either or both advanced forms [2,3,4]. Loss of central vision from the progression of atrophy can take years, whereas that from neovascular AMD can occur within a few months [5,6,7]. Most eyes with neovascular AMD develop vision loss, but quiescent (non-exudative, inactive) neovascularization can exist beneath the retinal pigment epithelial (RPE) monolayer without reducing visual acuity [8,9,10,11,12]. Vision loss from neovascular AMD often occurs from the invasion of endothelial cells from the choroid into the neural retina [13,14], where they are joined by other cell types to proliferate into neovascular lesions, known as type-2 macular neovascularization (MNV) [15]. Therefore, it is important to understand the molecular mechanisms that mediate choroidal endothelial cell invasion into the outer retina in order to identify safe and effective treatments that do not remove vascular support of the outer retina.

AMD is related to aging, diet, and smoking, but occurs late in life, despite strong genetic associations [16,17,18,19,20]. External stresses associated with aging are believed to increase oxidation, inflammation, and angiogenesis [21]. When external stresses overwhelm homeostasis, pathologic events occur. Some ways that this occurs are through cross-talk among growth factor-mediated signaling events and feed-forward loops involving common effectors in cell-signaling. Although treatments with anti-angiogenics that interfere with the bioactivity of vascular endothelial growth factor (anti-VEGF) have revolutionized outcomes in neovascular AMD, about 50% of patients continue to experience vision loss [22]. This article reviews science regarding the regulation of signaling cascades involved in choroidal endothelial cell invasion of the outer retina.

**Figure 1:**
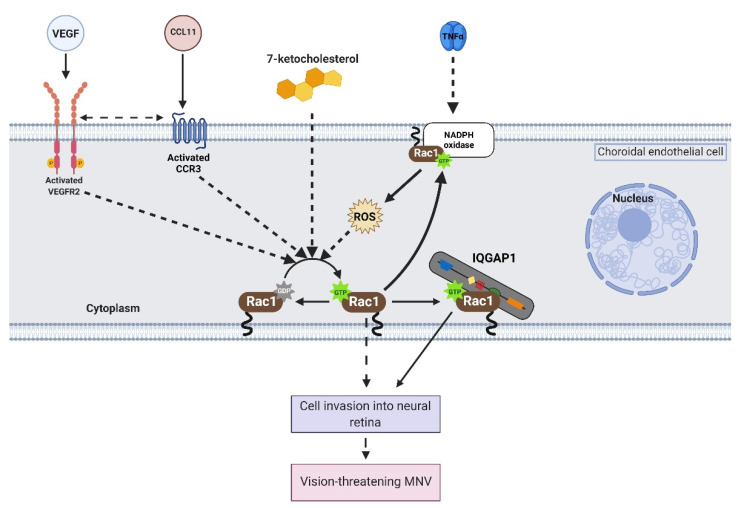
Cross-talk and feed-forward signaling activate Rac1 in choroidal endothelial cells. Vascular endothelial growth factor (VEGF) binds to VEGF receptor 2 (VEGFR2) and activates the receptor tyrosine kinase while C-C motif chemokine 11 (CCL11), an angiogenic eosinophil chemotactic protein, binds and activates signaling through the G protein-coupled receptor, C-C chemokine receptor 3 (CCR3). Each leads to activation of Rac1 (Rac1GTP) and, together, synergistically exacerbate Rac1 activation. Tumor necrosis factor alpha (TNFα) leads to activation of nicotinamide adenine dinucleotide phosphate (NADPH) oxidase, and the reactive oxygen species (ROS) generated activate Rac1, which can feed-forward to activate NADPH oxidase. 7-ketocholesterol (7KC) increases Rac1 activation. Activated Rac1 binds the GTPase related domain (GRD, dark green oval domain) of IQ motif containing GTPase activating protein 1 (IQGAP1) and has sustained activation. Dashed black lines represent indirect interactions, and solid black lines represent direct interactions (image created with BioRender.com).

Endothelial cell migration involves dynamic actin cytoskeletal rearrangements that promote the formation of a leading edge highlighted by cell protrusions (i.e., lamellipodia and filipodia) and the retraction of the trailing edge [23]. This process is regulated by several effectors downstream of different signaling cascades (i.e., Rho family of GTPases, PI-kinases, Ca^2+^/calcineurin, as examples). To identify effectors involved in choroidal endothelial cell migration, we developed a physiologically relevant human coculture assay using choroidal endothelial cells and RPE cells to recapitulate events surrounding choroidal endothelial cell transmigration of the RPE monolayer, a necessary step in type-2 MNV [24]. Choroidal endothelial cells that were cocultured in contact with the basal aspect of an RPE cell monolayer had significantly increased, Ras-related C3 botulinum toxin substrate 1 (Rac1) activation compared to solo cultured or cocultured with non-RPE cells as controls [25]. Rac1 is a member of the Rho family of GTPases that cycles from active to inactive states (see Section 2). Inhibiting endogenous active Rac1-mediated signaling in choroidal endothelial cells by transduction with either green fluorescent protein (GFP)-tagged dominant negative Rac1 or a GFP-tagged p21-activated kinase binding domain (PBD) protein reduced migration across the RPE cell monolayer compared to choroidal endothelial cells transduced with GFP as control [25]. Studies have since demonstrated that Rac1 is activated in choroidal endothelial cells by several AMD-associated stresses, tumor necrosis factor alpha (TNFα) [26], an example of an inflammatory cytokine; vascular endothelial growth factor (VEGF) [27,28,29,30,31,32] or C-C motif chemokine 11 (CCL11) [30], angiogenic stimuli; reactive oxygen species (ROS) [26]; and 7-ketocholesterol (7KC) [29,33], an oxidized cholesterol that accumulates in human Bruch’s membrane (Figure 1). A study also demonstrated CD93, a transmembrane glycoprotein [34] that is overexpressed in endothelial cells within choroidal neovascular membranes [35,36], was necessary for Rac1 activation and migration in human umbilical vein endothelial cells (HUVECs) [37]. Overall, the data support the idea that active Rac1 is an important downstream effector of AMD-associated stresses. Therefore, the focus of this review article is to discuss molecular mechanisms that regulate pathologic Rac1 activation in endothelial cells. This information may help to identify targeted therapeutic approaches that reduce activation and the invasive quality of choroidal endothelial cells without inhibiting vascular support of the outer retina, thereby inhibiting neovascular AMD and potentially reducing atrophic AMD.

## 2. Activation of Rac1 GTPase in Endothelial Cells

**Figure 2:**
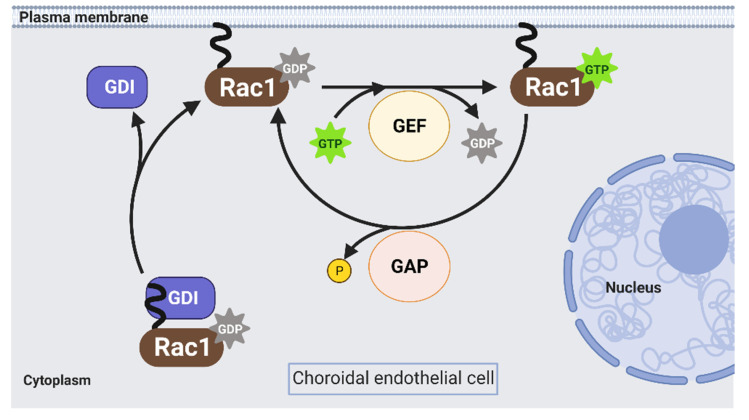
Rac1 GTPase acts as a biologic switch in choroidal endothelial cells. Rac1 GTPase cycles from an inactive, guanosine diphosphate (GDP)-bound, state to an activated, guanosine triphosphate (GTP)-bound, state. The primary regulators of Rac1 activation are GDP dissociation inhibitors (GDIs), guanine nucleotide exchange factors (GEFs), and GTPase activating proteins (GAPs). GDIs prevent the translocation of inactive Rac1 to the plasma membrane by interacting with lipid moieties on Rac1 (e.g., farnesyl or geranylgeranyl lipids). GEFs activate Rac1 by replacing bound GDP on Rac1 with GTP. GAPs inactivate Rac1 by accelerating the hydrolysis of bound GTP on Rac1 to GDP. Solid black lines represent direct interactions (image created with BioRender.com).

Rac1 is an approximate 21 kDa GTPase that is part of the Rac subfamily of the Rho family of GTPases and is the most-studied isoform in endothelial cells. In response to various stimuli, Rac1 cycles from an inactive GDP-bound form (Rac1GDP) to an active GTP-bound form (Rac1GTP) to transduce signaling intracellularly. The biologic switch is primarily modulated by three proteins: Rho guanine nucleotide exchange factors (Rho GEFs), Rho GTPase activating proteins (Rho GAPs), and Rho GDP dissociation inhibitors (Rho GDIs) (Figure 2).

### 2.1. Rho GEFs

Rho GEFs enable Rac1 activation by exchanging bound GDP on the GTPase with GTP. There are two classes of Rho GEFs: the Dbl family and the dedicator of cytokines (DOCK) family of proteins. Studies report the expression of both classes in endothelial cells [38]. For example, human microvascular endothelial cells (HMECs) express 47 Dbl family Rho GEFs, of which 17 have conserved amino acid residues that are known to interact with Rac1 [39]. Specifically, a study found that knockdown of Vav2, a member of the Dbl family, significantly reduced VEGF-induced Rac1 activation in HUVECs and human dermal microvascular endothelial cells [40]. Another study identified mRNA expression of 21 members of the Rho GEF family in HUVECs, of which nine are known to activate Rac1 [38]. Studies have also demonstrated that DOCK1, DOCK4, and DOCK9 activate Rac1 in other types of endothelial cells [41,42,43,44]. In choroidal endothelial cells, pre-treatment with a pharmacologic Rac1 inhibitor that prevents Rac1GDP interactions with the Dbl family Rho GEFs, TRIO and TIAM, reduced Rac1 activation induced by 7KC [33]. In parallel experiments, pre-treatment with the Rac1 inhibitor reduced choroidal endothelial cell migration in response to VEGF [32] or 7KC [33].

### 2.2. Rho GAPs

Rho GAPs facilitate the inactivation of Rac1 through the hydrolysis of Rac1GTP to Rac1GDP. In HUVECs, the mRNA expression of 17 different Rho GAPs were identified, and seven of these are known to inactivate Rac1 [38]. Studies demonstrated that overexpression of Rho GAPs in HUVECs reduced Rac1 activation, which was associated with reduced proliferation, migration, and tube formation [45,46]. The expression and role of Rho GAPs in choroidal endothelial cells have yet to be determined.

### 2.3. Rho GDIs

Rho GDIs also prevent Rac1 activation by binding directly to Rac1GDP and preventing GEF-mediated exchange at the plasma membrane [47,48]. However, HUVECs transfected with a point mutant Rac1 construct, R66A, which decreases Rac1 binding to Rho GDI, did not increase active Rac1-mediated lamellipodia formation compared to transfection with a wild-type Rac1 construct [49]. This finding suggests that the interaction between Rac1 and Rho GDI is complex and additional studies are required to better understand the role of Rho GDIs in endothelial cells.

### 2.4. Limitations of Regulating Rac1 Activation by Targeting Rho Gefs, Gaps and Gdis

Taken together, the data suggest that inhibiting Rac1 activation by interfering with Rac1GDP interactions with Rho GEFs might be a therapeutic option for neovascular AMD. However, inhibitors against Rac1-specific GEFs are inefficient [50,51], perhaps due to the expression of diverse members of the Rho GEF family. Also, the use of current Rac1 inhibitors is not possible due to the high concentration of the drug required to effectively block Rac1 activation [52]. We predict that overexpression of Rho GAPs in choroidal endothelial cells would also reduce Rac1 activation and choroidal endothelial cell migration. Although pharmacologic activation or overexpression of Rho GAPs can decrease Rac1 activation and prevent choroidal endothelial cell activation, there is no information regarding the clinical feasibility of this approach. Even if pharmacologic drugs were used to activate Rac1-specific GAPs, they may not be a viable therapeutic approach due to potential concerns of inhibiting physiologic roles of Rac1 activation (see below, Section 3). Similarly, overexpression of Rho GDIs might reduce Rac1 activation in choroidal endothelial cells; however, the concern of reducing physiologic Rac1 activation persists [53]. Overall, preventing Rac1 activation by targeting its primary regulators might not be a preferred therapeutic approach to prevent pathologic choroidal endothelial cell activation. Therefore, identifying alternative ways to regulate pathologic Rac1 activation is warranted. A better understanding of downstream Rac1GTP-mediated signaling might help to identify alternative approaches to specifically reduce the pathologic effects of Rac1GTP.

## 3. Activated Rac1-Mediated Effectors in Endothelial Cells

Once activated, Rac1 interacts with numerous proteins to promote endothelial cell migration. Some well-studied examples include members of the p21-activated kinases (PAKs) family, nicotinamide adenine dinucleotide phosphate (NADPH) oxidase (NOX) isoforms, and IQ motif containing GTPase activating proteins (IQGAPs).

### 3.1. PAKs

PAKs are serine/threonine kinases that are activated by interacting with RhoGTPases, including Rac1GTP [54]. Based on structural homology similarities, PAKs are classified as either group 1 (PAKs 1, 2, and 3) or group 2 (PAKs 4, 5, and 6). There have been several studies that provide insights into the role of PAKs in endothelial cells. One study demonstrated reduced growth medium-induced migration of HMECs that were co-injected with a construct expressing GFP, as a reporter to visualize cells, and a construct expressing dominant negative PAK that prevents endogenous PAK translocation to the plasma membrane, compared to GFP co-injected with wildtype PAK [55]. In a follow-up study, the authors developed a dominant-negative PAK peptide to determine the role of PAK in vivo. They found reduced basic fibroblast growth factor (bFGF)-induced angiogenesis in chick chorioallantoic membranes treated with the dominant negative PAK peptide compared to the control peptide [56]. In another study, compared to the control peptide, HUVECs pretreated with the dominant negative PAK peptide had reduced VEGF-induced β-catenin reorganization [57], a key step in endothelial cell migration. Crystal structures of PAK demonstrate that the protein is auto-inhibited by forming dimers with one another, and this process is disrupted by Rac1GTP interacting with PAK [58,59]. Therefore, a potential approach of preventing Rac1GTP-mediated choroidal endothelial cell migration might be to interfere with Rac1GTP interactions with PAK, and further testing can be considered in choroidal endothelial cells.

### 3.2. NADPH Oxidase (NOX)

The NOX family is comprised of seven isoforms; however, only NOX1 and NOX2 contain active Rac1 as a subunit [60]. NOX1 and NOX2 are expressed in endothelial cells [61,62] (see review on activation of endothelial NOX and NOX-mediated signaling pathways [63]); therefore, this subsection will focus on active Rac1-mediated NOX activation in choroidal endothelial cells and the development of experimental choroidal neovascularization (CNV) using the rodent laser-injury model, which represents aspects of MNV in humans, since rodents lack maculae [64].

p22phox, a subunit of both NOX1 and NOX2, and p47phox, a subunit of NOX2, are expressed in choroidal endothelial cells [26,27]. Compared to vehicle control, choroidal endothelial cells pretreated with diphenyleneiodonium, a NOX inhibitor, had significantly reduced VEGF-induced ROS generation assessed by 2′,7′-dichlorofluorescein (DCF)–diacetate (DA) fluorescence (DCFDA assay) [27]. In a separate study, knockdown of p22phox by siRNA in choroidal endothelial cells reduced TNFα-induced ROS generation compared to control siRNA [26]. These findings suggest that NOX subunits are not only expressed in choroidal endothelial cells but also sources of ROS generation in response to different age-related stresses. VEGF-induced ROS generation was reduced in choroidal endothelial cells transfected with Rac1 siRNA compared to control siRNA [27]. In addition, TNFα-induced Rac1 activation was reduced in choroidal endothelial cells transfected with p22phox siRNA compared to control siRNA [26]. These findings support the notion that active Rac1 is an important subunit in NOX-generated ROS that feed-forward to further activate Rac1 in choroidal endothelial cells. In parallel experiments, pretreatment with diphenyleneiodonium reduced VEGF-induced choroidal endothelial cell migration [27], whereas pretreatment with apocynin, an intracellular ROS quencher, reduced choroidal endothelial cell migration in response to VEGF [27] or TNFα [26]. The in vitro results were corroborated in vivo using the murine laser-induced CNV model, in which laser-induced CNV volume was reduced in mice that were treated with intravitreal apocynin compared to littermates that were treated with vehicle control. In addition, laser-induced CNV volume was also reduced in p47phox knockout (*Ncf1*^−/−^) mice compared to littermate wild-type mice [27]. Taken together, the data support the idea that NOX-mediated ROS generation is important for active Rac1-mediated choroidal endothelial cell migration and MNV by AMD-related stresses.

Although evidence suggests the inhibition of NOX-mediated ROS generation as a potential therapy, other studies have demonstrated that NOX-mediated ROS generation is also required for proper re-vascularization following injury [65,66]. To be functional, intravitreal administration of NOX inhibitors need to access intracellular signaling events. Moreover, intracellular inhibition of NOX-mediated ROS generation might interfere with the ability of macrophages to fight off infectious microbes.

### 3.3. IQGAPs

There are three known isoforms of IQGAPs; however, literature supports IQGAP1 and IQGAP2 expression in endothelial cells. IQGAP2 mRNA expression was elevated in human renal glomerular endothelial cells (HRGECs) by RT-PCR [67], and IQGAP2 was expressed in RPE/choroid lysates from *Iqgap1*^−/−^ mice and littermate *Iqgap1*^+/+^ mice [32]; however, the function of IQGAP2 in HRGECs and endothelial cells isolated from human RPE/choroids have yet to be determined. On the other hand, IQGAP1 is the most well-studied isoform in endothelial cells, including in choroidal endothelial cells. IQGAP1 is an approximate 190 kDa multidomain, scaffolding protein that binds to activated proteins and integrates signaling cascades to enable a cell to undergo a biologic function, such as cell migration [68,69]. In HUVECs, Yamaoka-Tojo et al. found increased immunofluorescent labeling of IQGAP1 at the leading edge of migrating HUVECs in response to VEGF; knockdown of IQGAP1 reduced VEGF-induced ROS generation measured in a DCFDA assay [70]. A follow-up study by these authors demonstrated immunofluorescent co-labeling of IQGAP1 and NOX2 at the leading edge of actively migrating HUVECs in a scratch injury assay [71]. In another study, knockdown of IQGAP1 by siRNA transfection prevented sphingosine-1-phosphate-induced migration of human pulmonary artery endothelial cells in a scratch injury assay [72]. As active Rac1 is an important subunit of NOX2 (see Section 3.2), these findings suggest that IQGAP1 may be necessary for the localization of active Rac1 to the plasma membrane during endothelial cell migration.

Wang et al. tested the role of IQGAP1 in Rac1-induced effects on choroidal endothelial cells [32]. IQGAP1 was identified in immunolabeled paraffin-embedded sections at the region of neovascular lesions in human donor eyes. IQGAP1 was expressed in lysates from cultured choroidal endothelial cells isolated from human donor eyes. In choroidal endothelial cells, knockdown of IQGAP1 by siRNA reduced VEGF-induced Rac1 activation and migration. The results were corroborated in vivo by using genetically modified mice in the laser-induced CNV model. *Iqgap1*^−/−^ mice had significantly reduced laser-induced CNV compared to littermate *Iqgap1*^+/+^ mice and reduced immunofluorescent co-labeling of Rac1GTP and lectin in sections of CNV lesions [32]. Since Wang et al. observed IQGAP1 protein expression in RPE/choroid lysates from wildtype mice, the authors bred commercially available mice to generate a tamoxifen-inducible Cre-loxP endothelial-specific IQGAP1 knockout mice along with littermate control mice that lack Cre recombinase and, therefore, continued to express endothelial IQGAP1 following tamoxifen administration. Tamoxifen-induced endothelial knockout of IQGAP1 in Cre-loxP mice resulted in significantly reduced laser-induced CNV and reduced Rac1GTP expression in cryosections of laser-induced CNV lesions when compared to tamoxifen-injected littermate controls [31]. Taken together, the in vivo data suggest that endothelial IQGAP1 is necessary for the development of CNV by interacting with active Rac1. On a molecular level, the GAP-related domain (GRD) of IQGAP1 binds to active Rac1 [73,74], and this interaction maintains Rac1 in its GTP-bound state instead of accelerating the hydrolysis of Rac1GTP to Rac1GDP due to the expression of threonine in place of the catalytic arginine [75,76]. Specifically, choroidal endothelial cells transfected with a GFP-tagged IQGAP1 construct that interfered with Rac1GTP binding IQGAP1 (GFP-IQ-MK24) [77] had reduced ability to sustain Rac1 activation by VEGF compared to choroidal endothelial cells transfected with control GFP-tagged full-length IQGAP1 (GFP-IQ-WT) [32]. Therefore, the data suggest that interfering with Rac1GTP binding to IQGAP1 might reduce choroidal endothelial cell activation, migration, and the development of invasive MNV.

There are several approaches to prevent effectors from binding to the domains of IQGAP1. For example, a study demonstrated the feasibility of developing cell-permeable peptides that disrupt domain-specific IQGAP1 interactions with other effectors [78]. The synthesis of a peptide that disrupts interactions between the GRD of IQGAP1 and Rac1GTP might prevent choroidal endothelial cell activation. Further studies are required to determine the possibility of this therapeutic approach in choroidal endothelial cells. IQGAP1 binds other protein partners, and studies reported that proteins can compete with one another to interact with IQGAP1 [79,80]. Of particular interest, calcium-induced calmodulin activation reduced Cdc42GTP interactions with IQGAP1 in a concentration-dependent manner [81], which reduced Cdc42 activation [82], suggesting that the competitive binding of effectors to IQGAP1 might reduce active Rac1-mediated choroidal endothelial cell migration (see Section 4).

## 4. Active Rap1a Interferes with Rac1 Activation in Choroidal Endothelial Cells

### 4.1. Activation of Rap1 Reduces CNV

Rap1 is a small, Ras-like GTPase that cycles from GTP-bound to GDP-bound states similar to Rac1. Rap1 is activated by GEFs (i.e., C3G, EPACs, PDZ-GEFs, RasGRPs, and DOCK4) [83] and inactivated by GAPs (i.e., Rap1GAP and SPA1) [84]. However, the activation of endothelial Rap1 by C3G and EPAC has been implicated in regulating barrier integrity following treatment with different stimuli [85]. For example, pretreatment with a pharmacologic activator of EPAC, 8CPT-2’OME-cAMP (8CPT), increased Rap1 activation and reduced thrombin-induced disruption of intercellular junctions in HUVECs, as visualized by β-catenin immunostaining [86]. In bovine retinal endothelial cells (BRECs), pharmacologic activation of EPAC by 8CPT also increased Rap1 activation and prevented VEGF-induced barrier compromise of the BREC monolayer assessed by measuring transendothelial electrical resistance [87]. These studies suggest that Rap1 activation might prevent endothelial cell migration by protecting barrier integrity. In line with this notion, 8CPT treatment significantly reduced choroidal endothelial cell migration across an RPE monolayer in the transmigration assay, and intravitreal 8CPT administration reduced lectin-stained CNV volume in rodent laser-induced CNV models [26,31,88,89]. 8CPT also increased Rap1 activation and reduced Rac1 activation by VEGF [31] or TNFα [26] in cultured choroidal endothelial cells. Although 8CPT is an EPAC-specific analog, EPAC can activate several downstream signaling effectors in addition to Rap1 [90]. Compared to adenoviral transduction with a vector that expressed GFP as control, choroidal endothelial cells transduced with adenoviral vectors that expressed GFP-tagged constitutively active Rap1a had significantly reduced Rac1 activation, migration, and tube formation induced by VEGF compared to PBS [31]. Adenoviral transduction of active Rap1a also significantly reduced choroidal endothelial cell Rac1 activation and ROS generation, measured by DCFDA fluorescence, in response to TNFα compared to PBS [26]. Taken together, the data suggest that Rap1 activation negatively regulates endothelial migration and angiogenesis by AMD-related stresses. However, HUVECs transfected with FLAG-tagged Rap1GAP1 vectors to inhibit Rap1 activation had significantly reduced VEGF-induced migration compared to transfection with an empty vector [91]. In contrast to no effect on Rap1 activation in choroidal endothelial cells treated with VEGF [31], murine lung endothelial cells demonstrated increased Rap1 activation when stimulated by VEGF [92]. These findings suggest that the role of Rap1 in angiogenesis is complex and may depend on the tissue and other signaling mechanisms [93]. However, data on choroid and choroidal endothelial cells support the line of thinking that activation of Rap1 antagonizes active Rac1-mediated choroidal endothelial cell migration and the development of MNV.

### 4.2. Active Rap1 Interacts with IQGAP1 to Interfere with Rac1 Activation in Choroidal Endothelial Cells

**Figure 3:**
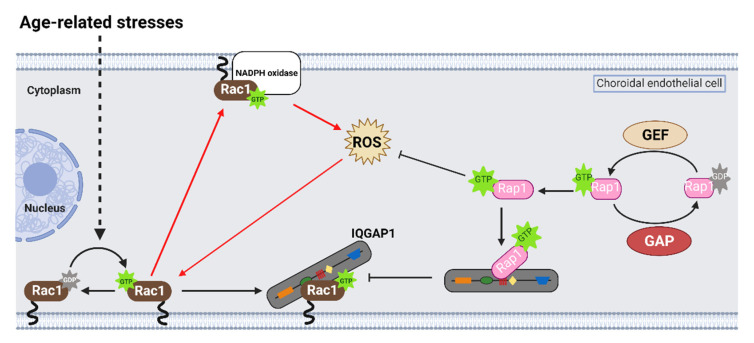
Active Rap1 antagonizes Rac1 activation by age- and AMD-related stresses in choroidal endothelial cells. Rap1 is a Ras-like small GTPase that cycles from an active, GTP-bound, state to an inactive, GDP-bound, state in choroidal endothelial cells. Active Rap1 (Rap1GTP) antagonizes Rac1 activation by age-related stresses, such as vascular endothelial growth factor (VEGF) or tumor necrosis factor alpha (TNFα). Active Rap1 binds to the IQ domain of IQGAP1 (red domain) and interferes with active Rac1 binding the GRD of IQGAP1 (dark green domain). Active Rap1 also interferes with reactive oxygen species (ROS) generation that further activates Rac1 by feed-forward signaling (red arrows). Dashed lines represent indirect interactions, and solid lines represent direct interactions (image created with BioRender.com).

Previous studies reported that active Rap1a binds to the IQ domain of IQGAP1 [94] and might displace effectors that bind to the other domains of IQGAP1 [80,95]. Therefore, active Rap1 interactions with the IQ domain might displace active Rac1 from the GRD and reduce pathologic choroidal endothelial cell activation and migration. To test this prediction, Ramshekar et al. used a mutant construct with point mutations in the IQ domain that resulted in enhanced Rap1 interactions with IQGAP1 [96]. Compared to a control construct, choroidal endothelial cells transfected with the mutant IQ construct had decreased VEGF-induced Rac1 activation and active Rac1 binding to IQGAP1 [31]. These findings suggest that active Rap1 antagonizing Rac1 activation involves a mechanism of interfering with Rac1GTP binding IQGAP1, a necessary step for sustaining Rac1 activation (Figure 3).

## 5. Conclusions and Future Directions

AMD is one of the leading causes of blindness worldwide. Treatment of neovascular AMD is successful in about 50% of patients with anti-VEGF agents, and there is no treatment to prevent the progression of atrophic AMD. Therefore, there is a need to identify novel treatments. The complicating factors in understanding the pathophysiology of AMD are the many stresses involved in AMD progression and the late development of advanced AMD despite genetic predisposition. Using the coculture model and the laser-induced CNV model, Rac1GTP, which is activated by AMD-related stresses, has been identified as an important regulator of choroidal endothelial cell activation and migration.

Rac1 has physiologic effects, making the broad inhibition of its activity infeasible. However, through binding IQGAP1 at the GRD domain, Rac1 has sustained activation and leads to pathologic choroidal endothelial cell activation and migration. Modulating IQGAP1 and Rac1GTP interaction might be a future method for AMD. The data also show that active Rap1a can interact with IQGAP1 and reduce sustained Rac1 activation, thereby reducing choroidal endothelial cell migration. The in vivo studies provide evidence that knockout of IQGAP1 or the pharmacological activation of Rap1 can reduce experimental CNV. Future studies to regulate Rac1 activation through pharmacologic or gene therapy approaches to activate Rap1 may prove valuable in treatments for AMD.
